# Defects in Regulation of Local Immune Responses Resulting in Atherosclerosis

**DOI:** 10.1080/17402520500182295

**Published:** 2005-09

**Authors:** Miroslav Ferenčík, Viera Štvrtinová, Ivan Hulín

**Affiliations:** 1 Institute of Immunology Faculty of Medicine Comenius University Sasinkova 4 Bratislava 811 08 Slovakia; 2 Institute of Neuroimmunology Slovak Academy of Sciences Sasinkova 4 Bratislava 811 08 Slovakia; 3 Clinic of Internal Medicine Faculty of Medicine Comenius University Bratislava Slovakia; 4 Institute of Pathophysiology Faculty of Medicine Comenius University Bratislava Slovakia

## Abstract

Atherosclerosis is nowadays generally accepted as an inflammatory disease but the mechanism of its origin and development have not yet been fully clarified. The present review focuses on the role of the local immune system as one of the key players in the pathogenesis of the complex process. Its part represented by vascular-associated lymphoid tissue (VALT) within the arterial wall participates directly in the vascular wall's homeostatis. Its inordinate activation during ontogenic development of an individual, this formerly defensive and physiologic mechanism transform into a pathological process resulting in an impairing inflammation. Hsp60, CRP and oxidized or otherwise modified LDL are serious candidates for triggering these pathological changes. The principal role is played by anti-Hsp60 antibodies and by shear stress originating on the surface of endothelium due to blood flow. The experimental and clinical data supporting this immunological hypothesis of atherosclerosis are discussed.


*Clinical & Developmental Immunology*, September 2005; 12(3): 225–234

Taylor & Francis

Taylor & Francis Group

REVIEW ARTICLE

### Defects in regulation of local immune responses resulting in atherosclerosis

MIROSLAV FERENČÍK[1,2], VIERA ŠTVRTINOVÁ[3], & IVAN HULÍN[4]

[1]*Institute of Immunology, Faculty of Medicine, Comenius University, Sasinkova 4, 811 08 Bratislava, Slovak Republic,* [2]*Institute of Neuroimmunology, Slovak Academy of Sciences, Bratislava, Slovak Republic,* [3]*Clinic of Internal Medicine, Faculty of Medicine, Comenius University, Sasinkova 4, 811 08 Bratislava, Slovak Republic, and* [4]*Institute of Pathophysiology, Faculty of Medicine Comenius University, Bratislava, Slovak Republic*

**Abstract**
Atherosclerosis is nowadays generally accepted as an inflammatory disease but the mechanism of its origin and development have not yet been fully clarified. The present review focuses on the role of the local immune system as one of the key players in the pathogenesis of the complex process. Its part represented by vascular-associated lymphoid tissue (VALT) within the arterial wall participates directly in the vascular wall's homeostatis. Its inordinate activation during ontogenic development of an individual, this formerly defensive and physiologic mechanism transform into a pathological process resulting in an impairing inflammation. Hsp60, CRP and oxidized or otherwise modified LDL are serious candidates for triggering these pathological changes. The principal role is played by anti-Hsp60 antibodies and by shear stress originating on the surface of endothelium due to blood flow. The experimental and clinical data supporting this immunological hypothesis of atherosclerosis are discussed.

### Introduction

Despite the fact that atherosclerosis has been subject to intensive research, its mechanisms of origin and development (atherogenesis) have not yet been fully clarified. Many cells and mediators that are typical representatives of inflammatory reactions participate also in atherosclerosis. Therefore, it is justified to assume that atherogenesis represents an inflammatory process (Ross 1999), the regulation of which is affected by many intervening risks. According to the American Heart Association, 268 such factors are currently known. This indicates that atherosclerosis is not a simple reaction but a complex process developing in course of many years. On the other hand, a serious doubt can be expressed as to whether all of these factors represent real risks of atherogenesis or whether many of them are rather triggering or initiating factors that steer latent processes and to a certain extent also physiologic processes into known pathological clinical signs, as, e.g. ischemic heart disease, myocardium infarction or a cerebrovascular stroke. This can be supported by the fact that only 50% of patients with the disease actually manifest hyperlipidemia, the popular major secondary risk factor targeted for intervention world/wide (EUROASPIRE 1997). Furthermore, the prevalence of traditional risk factors such as smoking, hypertension and diabetes is only slightly higher in patients with documented cardiac or peripheral vascular disease compared to similarly elderly patients without disease (Greenland et al. 2003). On the other hand, a significant percentage of aged people who have never developed clinical cardiovascular symptoms and died due to other reasons have developed progressive atherosclerosis of major and medium arteries.

It seems that, so far the only indubitably documented process taking place in coincidence with the origin and development of atherosclerosis is the remodelling of arterial vascular wall and local chronic inflammation. It is the immune system that participates in remodelling and all forms of inflammation. The inflammation is a phylogenetically and ontogenetically the oldest mechanism of innate immunity, the primary function of which is a protective reaction in response

Correspondence: M. Ferenčík, Institute of Immunology, Faculty of Medicine, Comenius University, Sasinkova 4, 811 08 Bratislava, Slovak Republic. E-mail: miroslav.ferencik@fmed.uniba.sk

ISSN 1740-2522 print/ISSN 1740-2530 online © 2005 Taylor & Francis
DOI: 10.1080/17402520500182295

to damage incurred to tissues and cells. Should this reaction be not sufficiently regulated, it may develop into impairing inflammation. In both protective and impairing types of inflammation, basic cells of the immune system are engaged, as neutrophils, macrophages or T lymphocytes, including endothelial and vascular smooth muscle cells (VSMC) in case of inflammation of vascular wall. These processes are affected by both pro-inflammatory and anti-inflammatory mediators released especially by participating cells, as, e.g. cytokines, prostanoids, adhesive and chemotactic molecules and C-reactive protein (CRP) (Langheinrich and Bohle 2005, Mullenix et al. 2005).

Should the inflammation be a natural response to the impairment of tissue, the knowledge of the mechanism, by which the vascular wall is impaired and subsequently afflicted by inflammation and atherosclerosis, is crucial. Out of several theories, trying to explain this mechanism, it is the autoimmunity theory that is of remarkable significance (Wick et al. 2001).

### Atherosclerosis as an inordinate activation of the immune system

Autoimmune diseases occur due to inordinate activation of the immune system and insufficiently regulated immune response. The reason resides in strong or long-term antigenic stimuli turning the original physiological and regulatory functions of autoimmune responses into auto-aggressive reactions.

The viability of the idea that atherosclerosis originates and develops on the basis of pathological autoimmune mechanism is indicated by two facts: (1) The process of atherosclerosis meets all of the determinant criteria worked out by Witebsky and Rose for including a disease unit into the category of autoimmune diseases. (2) In some classic autoimmune diseases as systemic lupus erythematosus (SLE), antiphospholipid syndrome (APS) or rheumatoid arthritis (RA) an accelerated progression of atherosclerosis is observed. In vasculitis, the vessel originally afflicted by inflammation tends to become liable to succumbing to atherosclerosis (Štvrtinová et al. 2001).

Criteria indicating that auto-aggressive reactions participate in the pathogenesis of atherosclerosis are as follows:

1. The presence of specific auto-antigens. In atherosclerosis, it is the group of heat shock proteins Hsp60/65, oxidized low-density lipoprotein (oxLDL) and $\beta_2$-glycoprotein-1 ($\beta_2$GP-1).
2. Active immunization in experimental animals. The immunization with Hsp60, oxLDL and $\beta_2$GP-1 induces the production of specific antibodies.
3. The evidence of the pathogenetic role of autoantibodies. Autoantibodies against Hsp60 and $\beta_2$GP-1 enhance the progression of atherosclerosis whereas autoantibodies against oxLDL have a protective function.
4. Passive transfer of disease by means of T lymphocytes. It has been proved that in mice the administration of specific anti-Hsp60 and anti-$\beta_2$GP-1 T lymphocytes induces the origin and development of atherosclerosis.
5. Immunomodulation therapy decreases the occurrence and intensity of experimental atherosclerosis in mice.

Among the above autoantigens, most significant are Hsp60 heat shock proteins being released from various cells of macroorganism during stress, or descending from some of bacterial species (chlamydia) and viruses. Other bacteria (mycobacteria) release an Hsp65 protein, which is similar to the former. The molecules of Hsp60 and Hsp65 descending from various sources greatly relate in their primary structure and that is why antibodies formed against Hsp60 of chlamydia react also with Hsp60 released from stressed cells of macroorganism. This reaction brings about the formation of immune complexes that activate the complement and trigger the inflammatory reaction (Wick et al. 2001). Patients with atherosclerosis yield increased levels of autoantibodies and increased counts of T lymphocytes aimed at heat shock proteins, especially at Hsp60, the fact of which might be of value in prognosis and diagnosis (Mandal et al. 2004).

It was also shown that immunization of mice with Hsp60 and with $\beta_2$-GPI served to enhance the progression of atherosclerosis and led to an increase in the infiltration of T lymphocytes in the subendothelial regions of the early plaques (Shoenfeld et al. 2000).

In addition to their key role in the origin of foam cells, oxidized LDLs have a direct cytotoxic effect on endothelial cells as well as a strong immunogenic impact. Due to the latter fact, autoantibodies of natural character are formed against them. Anti-oxLDL antibodies have a protective effect on the formation of foam cells, because they slow down the interaction of oxLDL with scavenger receptors on the surface of monocytes, macrophages and smooth muscle cells. On the other hand, their immune complexes on the surface of endothelial cells stimulate the inflammatory reaction (Gordon et al. 2001, Frostegard 2002).

The incidence of cardiovascular diseases is 50-fold higher in women suffering from SLE than in those not suffering from the latter disease (Manzi et al.

1999). Similar increases in the progress of atherosclerosis and their subsequent manifestations have been found in people suffering from antiphospholipid syndrome (George and Shoenfeld 1997, Gordon et al. 2001).

A thorough investigation carried out in recent years has shown that in addition to autoimmune mechanisms represented by autoantibodies and specific T lymphocytes, there are also other parts of the immune system that participate in atherosclerosis.

### Immunological hypothesis of atherosclerosis

The current immunologic hypothesis of the origin and development of atherosclerosis is based on the assumption that its core resides in insufficiently regulated local immune response.

It mainly implies from immunohistologic observations of very early clinically latent arterial changes. It has been revealed that the primary cells appearing in arterial intima in sites predetermined for atherosclerotic lesions are not the foam cells, but the lymphoid cells followed by macrophages and smooth muscle cells (Xu et al. 1990). Out of lymphoid cells, especially the helper ($CD4^+$) T lymphocytes are significantly more abundant than cytotoxic ($CD8^+$) T lymphocytes. Out of individual subpopulations of $T_H$ lymphocytes it is the subpopulation of $T_H1$ lymphocytes that prevails. As to their phenotype, they are focused on the secretion of pro-inflammatory cytokines (TNF, IFN-$\gamma$ and IL-2). Such an infiltration of arterial intima with mononuclear cells occurs as early as in one-year-old children. Up to 8–10 years of age neither foam cells nor extracellular LDL deposits are present (Millonig et al. 2002). In addition to T lymphocytes, macrophages and smooth muscle cells, normal arterial intima contains also scattered mast cells and a significant amount of immature forms of dendritic cells (Bobryshev and Lord 1999, Bobryshev 2000). They are present not only in intima, but also in adventitia. They mature later during the development of atherosclerosis.

The stimuli enhancing atherosclerosis including oxLDL increase the adhesion of dendritic cells to the endothelium and their migration into the intima (Alderman et al. 2002). After endothelial transmigration they settle in sites where arteries are exposed to the main hemodynamic stress. In these sites, they can participate in the development of atherosclerosis.

Infiltrations of mononuclear cells in intima, primarily in sites predetermining the origin of atherosclerotic lesions form vascular-associated lymphoid tissue (VALT) (Waltner-Romen et al. 1998). VALT can be considered to be an analogue of MALT, i.e. mucose-associated lymphoid tissue. It is true, however, that the local accumulation of cells in MALT is significantly more extensive than in VALT. MALT represents the local immune system of mucose. Its primary task is to carry out local immune responses. It is therefore, possible to assume that VALT has also a similar function. The latter statement is supported by the fact that VALT is present in all people regardless of age. In children up to 10 years of age, the cells of VALT are mostly in inactive state and can be considered to be natural structures. However, VALT in young Americans with no symptoms of cardiovascular diseases already at the age of 15–34 years contains cells yielding some signs of proinflammatory activities (Millonig et al. 2002).

The cells infiltrating the intima under physiological conditions as well as in progressive atherosclerosis are part of mechanisms of natural and acquired immunity.

### The role of innate immunity in atherosclerosis

Multi-cellular organisms including humans have developed and currently exist amidst masses of microorganisms. Therefore, on their own cells they had to develop biosensors enabling them to be recognized from those of microbes. This recognising system not only initiates the liquidation of pathogenic microbes that have penetrated into the organism, but also prevents their genetic material from combining with that of the cells of macroorganism. This system participates in innate immunity mechanisms. It is based on highly conserved pattern-recognition receptors (PRRs). They occur especially on cells that come into contact with external surroundings and pathogens as well as on VALT cells. PRRs are composed of two basic groups:

1. Receptors facilitating phagocytosis, i.e. opsonin receptors, as, e.g. receptors of immunoglobulin Fc-domains (especially $Fc_{\gamma}R$), C3b fragment of complement (CR1, CR3) and lectin receptors.
2. Toll-like receptors: 11 of them are known and referred to as TLR1–TLR11. TLRs recognize pathogen-associated molecular patterns (PAMPs).

This mechanism enables the several dozens of receptors to recognize molecular patterns of pathogens occurring in thousands of microorganisms' species within our environment. This is enabled by the fact that a particular pathogen pattern occurs in several species of microorganisms. Lipopolysaccharide (LPS) occurring on the surface of many gram-negative bacteria can serve as an example of pathogen pattern, as well as lipoteichoic acid, which is a characteristic molecular pattern of gram-positive bacteria. PRRs, however, do not recognize only molecular patterns occurring on the surface of microorganisms but also pathologically changed molecules (especially proteins) descending from the cells belonging to the macroorganism. These originate in various stress situations, as oxidation stress, presence of heavy metals and analogues of amino acids, heat, ionising radiation, etc. This means that they are the actual sensors of risks endangering the homeostasis of organism (Medzhitov 2001, Vink et al. 2004).

When PPR on the surface of T lymphocytes, macrophages, endothelial or smooth muscle cells in the vascular wall recognizes a particular PAMP, the protective inflammation is initiated. In case that the presence of such PAMP persists, the inflammation becomes chronic and incurs damage. The latter facts imply that the initiating stimuli can be endogenous as well as exogenous factors, while their number can be relatively large.

One of them is oxLDL, which contains a denatured protein representing the pathogenic pattern. Therefore, it is a specific ligand for PRRs (especially for CD14/TLR4 and CD36 receptors) on cells occurring in VALT. Already in small concentrations these cells stimulate the protective inflammatory reaction. If the presence of oxLDL persists, the protective inflammation turns into damage-incurring reaction. This can be one of early initiating factors of atherogenesis. At an increased concentration of oxLDL, the latter by means of scavenging receptors penetrates into monocytes, macrophages, smooth muscle and dendritic cells that turn into foam cells typical for the progressive stage of atherosclerosis.

The earliest effects of oxLDL include also the stimulation of expression of VCAM-1, vascular cell-adhesion molecule on endothelial cells of arteries. VCAM-1 is the crucial adhesion and chemotactic molecule for monocytes and T lymphocytes. In addition to the latter, oxLDL have a toxic effect on endothelial cells and a mitogenic impact on macrophages and smooth muscle cells (Wick et al. 2004). These properties allow oxLDL to be evaluated as one of the first initiating factors of atherogenesis as it stimulates not only the pro-atherogenic change of endothelial cells, but also the migration of monocytes and macrophages into intima and proliferation of smooth muscle cells.

In addition of oxLDL, PRRs recognize also other pathogenic patterns, namely those originating in injured cells of macroorganism. From the aspect of atherogenesis, the most important are the heat shock proteins with their relative molecular weight of 60 000—Hsp60 (Mandal et al. 2004). The family of Hsp60 contains highly conserved proteins, the amino acid composition of which is very similar to those of bacteria as well as of mammals including man. This enables various immunologic cross reactions to take place among autologous and microbial Hsp60. Hsp60 produce cells in result of the impact of various stressors. For example, human endothelial cells express Hsp60 in result of mechanical or heat stress, oxygen radicals, toxins, heavy metals, infectious agents, inflammatory cytokines, etc. (Xu and Wick 1996). Hsp60 are typical mitochondrial proteins and under the condition of stress they migrate to the surface of cells. That is why their appearance on the cellular surface is the manifestation of danger and represents a pathological pattern recognized by TLR-4 and -6 (Chen et al. 1999, Habich et al. 2002).

Atherogenesis also involves several components of humoral innate immunity as, e.g. natural antibodies, complement, CRP, mataloproteinases and their inhibitors (Wick et al. 2004).

### The task of acquired immunity in atherogenesis

Mechanisms of acquired immunity participate in atherogenesis by means of antibodies and T lymphocytes. Antibodies against autologous or exogenous antigens localized within the arterial wall form immune complexes with the latter. These complexes activate the complement, which injures the surrounding cells and triggers the inflammatory reaction. At the same time endothelial cells are activated. They express adhesion molecules to an increased extent, by means of which monocytes and other blood cells participating in the atherosclerotic process can adhere to the endothelium.

T lymphocytes occurring in human atherosclerotic plaques are of polyclonal origin. This means that at the time when they get into the plaques they are either already in an activated state or they become activated in the plaque by means of several antigens or cytokines (Stemme et al. 1991). The plaques contain more $T_H1$ lymphocytes producing typical pro-inflammatory cytokines (TNF-α, IFN-γ, IL-2) than $T_H2$-lymphocytes releasing anti-inflammatory IL-4, IL-5 and IL-10. Pro-inflammatory cytokines activate the local macrophages and inhibit the proliferation of smooth muscle cells of vessels. Due to the latter facts, the risk of plaque instability and rupture increases. As opposed to the latter, the anti-inflammatory cytokine TGF-β secreted by macrophages, $T_H3$-lymphocytes and smooth muscle cells have a pro-fibrotic effect and contribute to the stability of plaques (Mallat et al. 2001). The role of lymphocytes can be indicated also by the fact that a loss of spleen being an important secondary lymphatic organ leads to a severe manifestation of atherosclerosis (Witztum 2002).

Atherogenesis can involve also immune reactions in response to some infectious agents as, e.g. *Chlamydia pneumoniae* and some viruses. At the same time, there exists a proportion between mortality and the amount of infectious agents that have infested a particular individual and the stage of atherosclerosis in his/her arteries (Epstein et al. 2000). This applies especially in case of infectious agents that release high concentrations of Hsp60/65. This can be proved by the fact that the level of autoantibodies and T lymphocytes focused against Hsp60/65 significantly increases in people with severe atherosclerosis (Mandal et al. 2004).

### Inflammatory mediators and atherosclerosis

Chronic inflammatory processes become evident by increased blood levels of several markers, including sedimentation of erythrocytes, leukocyte and neutrophil counts, CRP level, α1-antitrypsine, ceruloplasmin, soluble adhesion molecules sICAM-1 (sCD54), sVCAM-1 (sCD106) and soluble E-selectin (sCD62E), sHsp60 and endotoxin (LPS). A significant coincidence with atherosclerosis has been found in cases of CRP, sHsp60, sVCAM-1, sCD62E, CD40-CD40L (CD154) interaction and LPS (Lind 2003).

However, some other mediators can participate in atherosclerosis. Some of them can have a stimulatory effect on its process whereas others inhibit them (Greaves and Channon 2002). The pro-atherogenic effect is induced by IFN-γ, IL-1β, IL-8 (chemokine CXCL8), IL-12, IL-18, MCP-1, (chemokine CCL2) and interaction CD40–CD154. The anti-atherogenic effect has been observed in coincidence with IL-4, IL-10, TGF-β and PPR-γ. In addition to typical cytokines, a significant pro-atherogenic effect has been proved in coincidence with some chemokines. In this sense, the most important is the monocyte chemoattractant protein MCP-1. The chemotactic activity on monocytes has been proved also in other chemokines of group CXCL, as, e.g. IL-8. Other chemokines occur in atherosclerotic plaques: CXCL10, $CX_3C$ (fractalkin), CCL11 (eotaxin), CCL17 and CCL22. Unfortunately, their precise roles in atherogenesis have not been clarified yet.

### *C-reactive protein*

The CRP is one of the earliest diagnostic inflammatory markers. Its concentration in blood serum increases several 100-fold in the case of inflammation when compared with normal levels. The limit of its detection by common methods is 5–10 mg/l, and these values used to be considered as normal. Currently, a highly sensitive method has been introduced decreasing this limit to 0.1 mg/l. By means of the latter, it is possible to assess high-sensitivity CRP (hsCRP). This fact has enabled the hsCRP to serve as the risk marker of atherosclerosis. This possibility is supported by epidemiologic studies (Ridger et al. 2002, Wang et al. 2002) showing that hsCRP values in people with cardiovascular diseases increase and signify the risk of coronary diseases, myocardium infarction, cerebrovascular stroke and cardiovascular death.

Currently, the values of 0.1–0.7 mg/l (yielded by 20% of population) are considered as normal; high values are those over 3.8 mg/l (20% of population). Individuals with values below 1 mg/l encounter a low risk of cardiovascular diseases; those with values of 1–3 mg/l are exposed to a moderate risk, whereas the values of 3–10 mg/l represent a high risk (Pearson et al. 2003, Libby and Ridger 2004).

However, CRP is a non-specific marker of inflammation and its concentration in chronic diseases as, e.g. in rheumatoid arthritis, infections, trauma or surgical interventions can significantly exceed the value of 3.8 mg/l. CRP values are increased in aged women, after hormonal replacement therapy as well as in diabetic and obese patients. These situations must be regarded when assessing the prognosis.

CRP is not only a prognostic marker of atherosclerosis, but it also participates in its pathogenesis. It can directly injure the vascular endothelium, induce atherosclerotic lesions including the mobilisation of macrophages and the formation of foam cells. Therefore, it is an important therapeutic target in primary prevention and therapy of diseases caused by atherosclerosis (Labarrere and Zaloga 2004).

### *Soluble Hsp60*

The sHsp60 increases significantly in patients with atherosclerosis located in carotid vessels and correlates with the thickness of arterial intima and media (Xu et al. 2000). It has been found out that the serum level of sHsp60 directly correlates with the titre of antibodies against Hsp60, LPS and chlamydia. In some patients, sHsp60 concentrations exceeded 1 mg/l. Increased sHsp60 concentrations have been found also in patients with hypertension and initial atherosclerosis (Pockley et al. 2000). The origin of sHsp60 is not known. Principally, it can be released from cells of human body after various physical, chemical, biological and psychological stressors or it can descend from infectious agents or released by inflammatory cells in effect of inflammatory cytokines. In addition to the latter, sHsp60 can descend from microparticles released from apoptotic and mechanically injured cells (Mallat and Tedgui 2001).

*CD40/CD40L interaction*

The CD40 is a receptor, whose ligand is CD40L (CD154). It occurs on the surface of many cells including endothelial and smooth muscle cells, T lymphocytes, macrophages and platelets as well as on those occurring in atherosclerotic plaques. CD40 is on the surface of activated T lymphocytes or in a soluble (sCD40L) form. The interaction between CD40 and CD40L induces a number of biochemical reactions and subsequent biologic manifestations of cells carrying CD40 molecules. It is applied also in atherogenesis, namely by increasing the expressions of intracellular metaloproteinases, pro-coagulation tissue factor, chemokines and inflammatory cytokines (Schoenbeck and Libby 2001a). When compared with membranous CD40, the soluble CD40 lacks a part of molecule. Despite the latter fact, biological activities of both are equal. The concentration of sCD40L in patients with cardiovascular diseases is increased and therefore it can be considered as a risk factor of prognostic value (Schoenbeck and Libby 2001b).

### Endothelial cells as a part of the immune system

In addition to basic functions in the regulation of cardiovascular system, vascular endothelial cells are also an important component of the immune system. In many ways, they are similar to macrophages as to their properties and their key role in initiating and developing the protective as well as impairing inflammatory responses. Endothelium can be considered as a particular transmission facility enabling bi-directional exchange of information between the cardiovascular and immune systems (Števrtinová et al. 1998). Mechanical or inflammatory impairments of endothelial integrity serve as initiating or triggering factors of atherosclerosis. They involve both hemodynamical and immune mechanisms. The impairment of endothelial monolayer results in dysfunction of endothelium or even apoptosis of endothelial cells. Both not only initiate these events, but also develop the process of atherosclerosis. Apoptosis results in the formation of apoptotic microparticles and in the need to replace the cells released from the endothelial layer.

The mechanical impairment or apoptotic destruction results in the formation of endothelial microparticles. The latter represent various fragments of endothelial cells. They represent about 30% of cellular microparticles that are present in circulation of healthy people. The rest of them are microparticles of platelets and leukocytes. Their amount increases in coincidence with various diseases including coronary syndromes (Diamant et al. 2004). These particles link with various inflammatory mediators, thus enabling their impact also beyond the site of their origin. They can settle in sites of injured endothelium, the fact of which can be proved by evidence that endothelial microparticles are the main components of atherosclerotic plaques.

Apoptosis of individual cells in endothelial monolayer takes place not only in result of impairment, but also in result of their normal biologic turnover, which is substantially higher than previously considered. In both cases, the missing cells are to be replaced. Such regeneration of endothelium can be carried out by means of circulating endothelial cells or by angiogenesis. It seems that the replacement by means of circulating endothelial cells is faster. Their origin is not precisely known. They can descend from bone marrow (progenitor endothelial cells), from tissue stem cells or from transformed blood monocytes (Dimmeler and Zeiher 2004). It is assumed that they play a key role in maintaining the integrity of endothelium and thus also in the prevention of atherosclerosis and thrombus complications. This is suggested also due to the fact that in patients with coronary diseases, the amount of circulating progenitor endothelial cells is significantly decreased (Vasa et al. 2001a). The reason of this decrease can reside in the depletion of stem and progenitor cells stored in bone marrow, their decreased mobilisation or decreased lifetime (biological half-time period).

Should the insufficient function of circulating progenitor endothelial cells really apply in the development of atherosclerosis and coronary disease, the pharmacologically induced increase in their amount should be of therapeutic significance. Such pharmacological intervention can take place by means of statins, which increase the amount and functional activity of circulating progenitor endothelial cells in experiment as well as in patients with stable disease of coronary arteries (Vasa et al. 2001b).

It is assumed that the optimal velocity of apoptosis of endothelial cells significantly determinates not only the maintenance of the integrity and normal function of endothelium, but also that of the whole vascular wall, because apoptosis of endothelial cells influences also the regulation of apoptosis of smooth muscle cells of vessels (Stoneman and Bennett 2004). An increase in apoptosis is the sign of impaired endothelium as well as that of its turning into pro-inflammatory and pro-atherogenetic phenotypes. In progressive atherosclerotic plaques, it is a sign of increased risk of their rupture. Stimulation of apoptosis is caused by classic risk factors of atherosclerosis, as, e.g. high level of blood glucose, increased oxidation stress, oxLDL and angiotensin II. Angioprotective factors as nitric oxide (NO) and shear stress have a local impact, and according to their levels they can either activate or suppress the apoptosis of endothelial cells.

One of the most important factors preserving the integrity of endothelium is the shear stress. It is a biomechanic force that is determined by blood current, viscosity and the geometry of vessel. The blood current within vessel can be of laminar or turbulent character. Shear stress has a direct impact on endothelium. It regulates its structures and biologic properties by means of mechanic transfer. Several molecules, structures and processes are involved in turning the mechanical stimuli put into effect by shear stress into biochemical signals resulting in regulated transcription of various genes within endothelial cells (Cheng et al. 2004). Their products directly regulate the structure and biologic functions of endothelial cells.

Laminar shear stress stimulates the expression of anti-inflammatory, anti-proliferation, anti-apoptotic and anti-oxidation genes. Proper maintenance of normal diameter of vessels, inhibition of proliferation of endothelial and smooth muscle cells and inhibition of thrombotic and inflammatory processes are determined by normal laminar shear stress, which in large arteries ranges from 5 to 20 $dyn/cm^2$ (Resnick et al. 2003). Should it drop below 5 $dyn/cm^2$, atherogenesis is stimulated because such a decrease provides mechanical and biochemical signals leading to decreased production of NO-synthase, inhibition of vasodilation and renewal of impaired endothelial cells. On the other hand, the decreased value of shear stress results in an increase in the production of reactive oxygen intermediates, permeability for lipoproteins, adhesion of leukocytes, apoptosis, proliferation of smooth muscular cells and deposition of collagen. At values over 40 $dyn/cm^2$, the shear stress mechanically injures the endothelium (Cunnigham and Gotlieb 2005). This means, that laminar shear stress must have a particular significance as to the maintenance of the accurate adherence of individual cells to inter-endothelial junctions and thus also to normal structure and physiologic properties of endothelium.

In some areas of the vascular bed, e.g. in the sites of their branching, bends or influx, the shear stress decreases, or in result of turbulent flow changes irregularly its intensity and direction resulting in increased apoptosis of endothelial cells located herein (Garcia-Cardena et al. 2001). Therefore, these locations are predetermined to be the prior sites of the origin of atherosclerotic lesions. An increased apoptosis of endothelial cells results also in an increase in the apoptosis of smooth muscle cells thus decreasing their presence in atherosclerotic plaques and increasing the risk of their rupture (Bennet 1999).

Non-laminar blood flow stimulates such particular changes in the expression of genes of endothelial cells, in their cytoskeleton structure and correcting mechanisms that turn endothelial cells into inflammatory and atherogenetic phenotypes. By means of intercellular communication, these changes are carried from endothelial cells over to smooth muscle cells. Between the expression of atheroprotective and pro-atherogenic genes within endothelial cells exists a fine balance that is regulated by the changes in shear stress. These can result in physiological or pathological remodelling of cells. The traditional risk factors as, e.g. long-term hypertension, smoking, hypercholesterolaemia and diabetes mellitus can disturb this balance (Landmesser et al. 2004). Hence, the critical function of shear stress resides in the regulation of normal physiological atheroprotective functions of vascular wall, as well as in the regulation of its pathological functions with subsequent complex changes enhancing the atherogenesis.

The decreased (insufficient) or irregular shear stress brings about an increase in apoptosis and subsequent dysfunction of endothelium. However, an extensively increased (more than two-fold of normal values) shear stress has also a negative impact by means of directly injuring the endothelium.

In result of apoptosis, the cells split into fragments—apoptotic bodies that are wrapped in the cytoplasmic membrane preventing the potential cytotoxic contents to leak into the surroundings and evoke inflammation. Apoptotic bodies are engulfed especially by surrounding macrophages. Should the velocity of this engulfment be insufficient, the bodies lose their membrane and release their content. The latter brings about an inflammatory reaction and apoptosis turns into necrosis. The engulfment of apoptotic bodies by macrophages is influenced by three factors: chemotactic movement of macrophages, the recognition and devouring of the particle. This mechanism plays the key role in the maintenance of tissue homeostasis of multi-cellular organisms. It is involved also in the homeostasis of vascular wall.

Three signals are involved in the latter: "find-me", "eat-me" and "don't-eat-me" (Lauber et al. 2004). Should the macrophage catch the signal of first type emitted by apoptotic cells, it begins to move chemotactically towards its source. In the course of its approximation, the eat-me signals are involved (various scavenging or lectin receptors). When the macrophage contacts the cell in the stage of its not being decided on apoptosis, it emits the do-not-eat-me signal preventing the macrophages from engulfment it.

Recently, the find-me type of signal has been partially clarified. It is assumed that this function is carried out by lysophosphatidyl choline (LPC), which is the main lipid compound of oxLDL (Lauber et al. 2003). The surface of macrophages is equipped by LPC receptors, the fact of which implies that LPC can function not only as a chemotactic factor emitted by apoptotic endothelial or other cells to the attention of macrophages and other professional phagocytes, but it can combine also with other scavenging receptors and enable the eat-me type of signal to be carried out. This fact could be especially important in the pathogenesis of atherosclerosis under the condition that LPC on apoptotic cells is equal to LPC in oxLDL, and their affinity for corresponding receptors on the surface of macrophages is comparable. In such cases, by means of partial occupation of LPC receptors and scavenging receptors on macrophages, oxLDL could slow down its movement and engulfment of apoptotic particles descending from endothelial and smooth muscle cells. A delayed liquidation of remnant apoptotic cells could be an impulse for triggering the inflammatory reaction resulting in the process of atherosclerosis.

### Conclusion

It is justified to assume that atherosclerosis just as other diseases begins as a single pathological triggering mechanism, which however, does not have to be equal in each individual. The determination of this primary event by using a simple diagnostic test would be very beneficial in prevention and effective therapy of vascular diseases caused by atherosclerotic processes. The experimental, epidemiologic and clinical knowledge gained in recent years indicates that one of the key players in the mechanism of atherosclerosis is the immune system. Its part represented by vascular-associated lymphoid tissue—VALT within the arterial wall participates directly in the local protection from exogenous as well as endogenous factors endangering the vascular wall's homeostasis. In the particular period of ontogenetic development of an individual, this formerly defensive and physiologic mechanism transforms into a pathologic process resulting in an impairing inflammation. Serious candidates for triggering this pathologic change are Hsp60, CRP and oxidized or otherwise modified LDL. Out of them, especially the serum concentration of soluble Hsp60, its specific antibodies and CRP could represent a simple diagnostic test indicating that a pathologic process is present in the vascular wall.

The principal role in the pathogenesis of atherosclerosis is played by shear stress originating on the surface of endothelium due to blood flow. By means of biochemical signals, its actual values influence the transcription of genes within endothelial cells and thereby also the intensity and direction of their biological responses being carried out not only in form of protective or impairing inflammation, but also in the form of structural remodelling.

During ontogenesis, the cardiovascular system overcomes morphologic changes, the objective of which is to make this system optimal not only in securing sufficient blood, but also in guaranteeing that its movement is within the optimal pressure range. The blood pressure gradually increases from childhood to adulthood due to the fact that the tension of vascular walls keeps changing and the volume of blood ejected by the heart increases whereas the amount of cardiomyocytes stays unchanged. These changes correspond with cellular remodelling. Each change, including physiological changes in blood pressure, is recorded by receptors on the surface of endothelial cells, where from the signal is transmitted either to the cellular nucleus where it influences the transcription of appropriate genes or to the cellular skeleton thus influencing the structure and shape of cell. The changes in the transcription of genes are reflected in anti- or pro-atherogenic phenotypes of endothelium, stimulation or suppressing its inflammatory responses. The changes in the cellular skeleton determine the vascular remodelling which represents the result of adaptation to actual pressure conditions. In the latter context, atherosclerosis could be considered as a regulatory remodelling failure in some sites resulting in the formation of atherosclerotic plaques. When using this approach, the participation of immune system by means of impairing inflammatory responses could be comprehended also as an actor in remodelling processes.

### References

Alderman CJJ, Bunyard PR, Chain BM, Foreman JC, Leake DS, Katz DR. 2002. Effect of oxidised low density lipoprotein on dendritic cells: A possible immunoregulatory component of the atherogenic micro-environment. Cardiovasc Res 55:806–819.

Bennet MR. 1999. Apoptosis of smooth muscle cells in vascular remodelling and atherosclerotic plaque rupture. Cardiovasc Fres 41:361–368.

Bobryshev YV, Lord RSA. 1999. 55 kDa actin-bundling protein (p55) is a specific marker for identifying vascular dendritic cells. J Histochem Cytochem 47:1481–1486.

Bobryshev YV. 2000. Dendritic cells and their involvement in atherosclerosis. Curr Opin Lipidol 11:511–517.

Chen W, Syldath U, Bellmann K, Burkert V, Kolb H. 1999. Human 60-kDa heat-shock protein: A danger signal to the innate immune system. J Immunol 162:3212–3219.

Cheng C, de Crom R, van Kaperen R, Helderman F, Gourabi BM, van Damme LCA, Kirschbaum SW, Slager CJ, van der Steen AFW, Kams R. 2004. The role of shear stress in atherosclerosis—action through gene expression and inflammation. Cell Biochem Biophys 41:279–295.

Cunnigham KS, Gotlieb AI. 2005. The role of shear stress in the pathogenesis of atherosclerosis. Lab Investig 85:9–23.

Dimmeler S, Zeiher AM. 2004. Vascular repair by circulating endothelial progenitor cells: The missing link in atherosclerosis? J Mol Med 82:671–677.

Diamant M, Tushuizen ME, Sturk A, Nieuwland R. 2004. Cellular microparticles: New players in the field of vascular disease? Eur J Clin Investig 34:392–408.

Epstein SE, Zhu J, Burnett MS, Zhou YF, Vercellotti G, Hajjar D. 2000. Infection and atherosclerosis: Potential roles of pathogen burden and molecular mimicry. Arterioscler Thromb Vasc Biol 20:1417–1420.

1997. EUROASPIRE: A European society of cardiology survey of secondary prevention of coronary heart disease: Principal results Eur Heart J 18:1569–1582.

Frostegard J. 2002. Autoimmunity, oxidized LDL and cardiovascular disease. Autoimmun Rev 1:233–237.

Garcia-Cardena G, Comanders J, Anderson KR, Blackman BR, Gimbrone, MA. Jr., 2001. Biomechanical activation of vascular endothelium as a determinant of its functional phenotype. Proc Natl Acad Sci USA 98:4478–4485.

George J, Shoenfeld Y. 1997. The anti-phospholipid (Hughes) syndrome: A cross roads of autoimmunity and atherosclerosis. Lupus 6:559–560.

Gordon PA, George J, Khamashta MA, Harats D, Hughes G, Shoenfeld Y. 2001. Atherosclerosis and autoimmunity. Lupus 10:249–252.

Greaves DR, Channon KM. 2002. Inflammation and immune responses in atherosclerosis. Trends Immunol 25:535–541.

Greenland P, Knoll MD, Stamler J, Neaton JD, Dyer AR, Garside DB, Wilson PW. 2003. JAMA 290:891–897.

Habich C, Baumgart K, Kolb H, Burkart V. 2002. The receptor for heat shock protein 60 on macrophages is saturable, specific, and distinct from receptors for other heat shock proteins. J Immunol 168:562–576.

Labarrere CA, Zaloga GP. 2004. C-reactive protein: From innocent bystander to pivotal mediator of atherosclerosis. Am J Med 117:499–507.

Landmesser U, Hornig B, Drexler H. 2004. Endothelial function—a critical determinant in atherosclerosis. Circulation 109:27–33.

Langheinrich AC, Bohle RM. 2005. Atherosclerosis: Humoral and cellular factors of inflammation. Virchows Arch 446:101–111.

Lauber K, Bohm E, Krober SM, Xiao YJ, Blumenthal SG, Lindemann RK, Marini P, Wieding C, Zobywalski A, Baksh S. 2003. Apoptotic cells induce migration of phagocytes via caspase-3-mediated release of a lipid attraction signal. Cell 113:717–730.

Lauber K, Blumenthal S, Waibel M, Wesselborg S. 2004. Clearance of apoptotic cells: Getting rid of the corpses. Mol Cell 14:277–287.

Libby P, Ridger PM. 2004. Inflammation and atherosclerosis: Role of C-reactive protein in risk assesment. Am J Med 116(Suppl. 1): 9–16.

Lind L. 2003. Circulating markers of inflammation and atherosclderosis. Atherosclerosis 169:203–214.

Mallat Y, Tedgui A. 2001. Current perspective on the role of apoptosis in atherothrombotic disease. Circ Res 88:998–1003.

Mallat Z, Gojova A, Marchiol-Fournigault C, Esposito B, Kamate C, Merval R, Fradelizi D, Tedgui A. 2001. Inhibition of tranforming growth factor-beta signaling accelerates atherosclerosis and induces an unstable plaque phenotype in mice. Circ Res 89:930–934.

Mandal K, Jahangiri M, Xu Q. 2004. Autoimmunity to heat shock proteins in atherosclerosis. Autoimmun Rev 3:31–37.

Manzi S, Meilah EN, Rairie JE, Conte CG, Medsger TA, JansenMcWilliams L, Dagostino RB, Kuller LH. 1999. Age specific incidence of myocardial infarction and angina in women with systemic lupus erythematosus: Comparison with the Framingham study. Am J Epidemiol 145:408–415.

Medzhitov R. 2001. Toll-like receptors and innate immunity. Nat Rev Immunol 1:135–145.

Millonig G, Malcom GT, Wick G. 2002. Early inflammatory-immunological lesions in juvenile atherosclerosis from the pathobiogical determinats of atherosclerosis in youth (PDAY)-study. Atherosclerosis 160:441–448.

Mullenix PS, Andersen CA, Starnes BW. 2005. Athrosclerosis as inflammation. Ann Vasc Sur 19:130–138.

Pearson TA, Mensah GA, Alexander RW, Anderson JKL, Cannon, RO, III, Criqui M, Fadl YY, Fortmann SP, Myers GL, Rifai N, Smith SC, Jr, Taubert K, Tracy RP, Vinicor F. 2003. Markers of inflammation and cardiovascular disease: Application to clinical and public health practice: A statement for health care professionals from the center for disease control and prevention and the american heart association. Circulation 107:499–511.

Pockley AG, Wu R, Lemme C, Kiessling R, de Faire V, Frostegard J. 2000. Circulating heat shock protein 60 is associated with early cardiovascular disease. Hypertension 36:303–307.

Resnick N, Yahav H, Shay-Salit A, Shushy M, Schubert S, Zilberman LCM, Wofowitz E. 2003. Fluid shear stress and the vascular endothelium: For better and for worse. Prog Biophys Mol Biol 81:177–199.

Ridger PM, Rifai N, Rose L, Buring JE, Cook NR. 2002. Comparison of C-reactive protein and low-density lipoprotein cholesterol levels in the prediction of first cardiovascular events. New Engl J Med 347:1557–1565.

Ross R. 1999. Atherosclerosis—an inflammatory disease. New Engl J Med 340:115–126.

Schoenbeck U, Libby P. 2001. The CD40/CD154 receptor/ligand dyad. Cell Mol Life Sci 58:4–43.

Schoenbeck U, Libby P. 2001. CD40 signaling and plaque instability. Circ Res 89:1092–1103.

Shoenfeld Y, Harats D, George J. 2000. Heat shock protein 60/65, β2-glycoprotein 1 and oxidized LDL as players in murine atherosceleros is. J Autoimmun 15:199–202.

Stemme S, Rymo L, Hansson GK. 1991. Polyclonal origin of T lymphocytes in human atherosclerotic plaques. Lab Investig 65:654–660.

Stoneman EA, Bennett MR. 2004. Role of apoptosis in atherosclerosis and its therapeutic implications. Clin Sci 107:343–354.

Štvrtinová V, Ferenčík M, Hulín I, Jahnová E. 1998. Vascular endothelium as a connecting operator in the information transfer between the cardiovascular and immune system. Bratisl Lek Listy 99:5–19.

Štvrtinová V, Rauová L, Tuchyňová A, Rovenský J. 2001. Vasculitis of the coronary arteries and atherosclerosis: Random coincidence or causative relationship?. In: Shoenfeld Y, Harats D, Wick G, editors. Atherosclerosis and autoimmunity. Amsterdam/Lausanne/New York/Oxford/Shannon/Singapore/Tokyo: Elsevier. p 315–327.

Vasa M, Fichtlscherer S, Aicher A, Adler K, Urbich C, Martin H, Zeiher AM, Dimmeler S. 2001. Number of migratory activity of circulating endothelial progenitor cells inversely correlate with risk factors for coronary artery disease. Circ Res 89:E1–E7.

Vasa M, Fichtlscherer S, Adler K, Mildner-Rihm C, Aicher A, Martin H, Zeiher AM, Dimmeler S. 2001. Increase in circulating endothelial progenitor cells by statin therapy in patients with stable coronary artery disease. Circulation 103:2885–2890.

Vink A, de Kleijn DPV, Pasterkamp G. 2004. Functional role for toll-like receptors in atherosclerosis and artderial remodeling. Curr Opin Lipidol 15:515–521.

Waltner-Romen M, Falkensammer G, Rabl W, Wick G. 1998. A previously unrecognized site of local accumulation of mononuclear cells: The vascular-associated lymphoid tissue. J Histochem Cytochem 46:1347–1350.

Wang TJ, Nam BH, Wilson PWF, Wolf PA, Levy D, Polak JF, D'Agostino RB, O'Donnell CJ. 2002. Association of C-reactive protein with carotid atherosclerosis in men and women: The Framingham heart study. Arterioscler Thromb Vasc Biol 20:1662–1667.

Wick G, Perschinka H, Millonig G. 2001. Atherosclerosis as an autoimmune disease: An update. Trends Immunol 22:665–669.

Wick G, Knoflach M, Xu Q. 2004. Autoimmune and inflammatory mechanisms in atherosclerosis. Annu Rev Immunol 22:361–403.

Witztum JL. 2002. Splenic immunity and atherosclerosis: A glimpse into a novel paradigm? J Clin Investig 109:721–724.

Xu Q, Wick G. 1996. The role of heat shock proteins in protection of pathophysiology of the arterial wall. Mol Med Today 2:372–379.

Xu QB, Oberhuber G, Gruschnitz M, Wick G. 1990. Immunology of atherosclerosis: Cellular composition and major histocompatibility complex class II antigen expression in aortic intima fatty streaks, and atherosclerotic plaques in young and aged human specimens. Clin Immunol Immunopathol 56:344–359.

Xu QB, Schett G, Perschinka H, Mayr M, Egger G, Oberhollenzer F, Willeit J, Kiechl S, Wick G. 2000. Serum soluble heat shock protein 60 is elevated in subjects with atherosclerosis in a general population. Circulation 102:14–20.

